# Platinum Nanoparticles Loaded Graphitic Carbon Nitride Nanosheets with Enhanced Peroxidase-like Activity for H_2_O_2_ and Oxidase-Based Sensing

**DOI:** 10.3390/molecules28093736

**Published:** 2023-04-26

**Authors:** Gege Yang, Ying Chen, Rui Shi, Rongrong Chen, Shanshan Gao, Xin Zhang, Yuan Rao, Ying Lu, Yuancheng Peng, Zhihe Qing, Chunxia Song

**Affiliations:** 1 Department of Applied Chemistry, School of Science, Key Laboratory of Agricultural Sensors, Ministry of Agriculture and Rural Affairs, Anhui Agricultural University, Hefei 230036, China; 2 Hunan Provincial Key Laboratory of Cytochemistry, Changsha University of Science and Technology, Changsha 410114, China; 3 School of Life Sciences, Anhui Agricultural University, Hefei 230036, China

**Keywords:** PtNP@g–C_3_N_4_ nanosheets, peroxidase-mimic, H_2_O_2_, oxidase-based sensing

## Abstract

Platinum nanoparticles (PtNPs) are classical peroxidase-like nanozyme; self-agglomeration of nanoparticles leads to the undesirable reduction in stability and catalytic activity. Herein, a hybrid peroxidase-like nanocatalyst consisting of PtNPs in situ growing on g–C_3_N_4_ nanosheets with enhanced peroxidase-mimic catalytic activity (PtNP@g–C_3_N_4_ nanosheets) was prepared for H_2_O_2_ and oxidase-based colorimetric assay. g–C_3_N_4_ nanosheets can be used as carriers to solve the problem of poor stability of PtNPs. We observed that the catalytic ability could be maintained for more than 90 days. PtNP@g–C_3_N_4_ nanosheets could quickly catalyze the oxidation of 3,3′,5,5′-tetramethylbenzidine (TMB), and the absorbance of blue color oxidized TMB (oxTMB) showed a robust linear relationship with the concentration of H_2_O_2_ (the detection limit (LOD): 3.33 μM). By utilizing H_2_O_2_ as a mediator, this strategy can be applied to oxidase-based biomolecules (glucose, organophosphorus, and so on, that generate or consume hydrogen peroxide) sensing. As a proof of concept, a sensitive assay of cholesterol that combined PtNP@g–C_3_N_4_ nanosheets with cholesterol oxidase (ChOx) cascade catalytic reaction was constructed with an LOD of 9.35 μM in a widespread range from 10 to 800 μM (R^2^ = 0.9981). In addition, we also verified its ability to detect cholesterol in fetal bovine serum. These results showed application prospect of PtNP@g–C_3_N_4_ nanosheets-based colorimetry in sensing and clinical medical detection.

## 1. Introduction

Since the first evidence of triazacyclonane-modified gold nanoparticles as substitutes for phosphodiesterase by Scrimin et al. in 2004 [1], especially due to the report of Fe_3_O_4_ nanoparticles with intrinsic peroxidase-like activity by Yan group in 2007 [2], nanomaterial-based artificial enzymes mimic, known as nanozymes, have received great research attention. Compared with natural enzymes, they possess the advantages of low cost, simple preparation, and tunable catalytic activity [3,4]. In recent years, various nanozymes including inorganic nanoparticles, carbon nanomaterials, metal-organic frameworks etc. have been used to mimic peroxidase, oxidase, and nuclease, et al. [5,6,7,8,9,10]. Particularly, ever-growing research interests have focused on peroxidase-mimic nanozymes owing to their application in sensing, catalysis, environmental protection, and other fields [11,12,13,14].

Among these nanozymes, platinum nanoparticles (PtNPs) are classical peroxidase-like nanozymes by virtue of their high chemistry stability and catalytic activity [15,16]. However, nanoparticles often self-aggregate during the catalytic reaction, thereby the active surface area is reduced, resulting in low stability and catalytic activity, which seriously limits the practical application of nanozymes [17,18]. In order to meet these challenges, we packaged platinum nanoparticles into a hyaluronidase-activated capsule and verified its high catalytic stability even under physiological conditions and antibacterial effect on diabetic wounds infected by bacteria [19]. A variety of nanosheets such as graphene, graphitic carbon nitride, and manganese dioxide were also used as support for inorganic nanoparticles decoration in order to hold preferable peroxidase-like performance, forming hierarchical nanohybrids [20]. Nanosheets could either offer synergistic effects on the intrinsic properties of the inorganic nanoparticles or prevent their aggregation, making the nanohybrids much more stable and attractive in catalytic sensing in comparison to the nanoparticles alone [21,22]. Graphite carbon nitride (g–C_3_N_4_) nanosheets have been intensely studied by virtue of their intrinsic peroxidase-like activity, excellent chemical stability, and appealing electronic structure [23]. Yang group [20] proved that the nanohybrid, with Au nanoparticles loaded on g–C_3_N_4_ nanosheets, possessed increased catalytic capability, and further constructed a colorimetric biosensing method based on its peroxidase-like activity.

**Figure 1:**
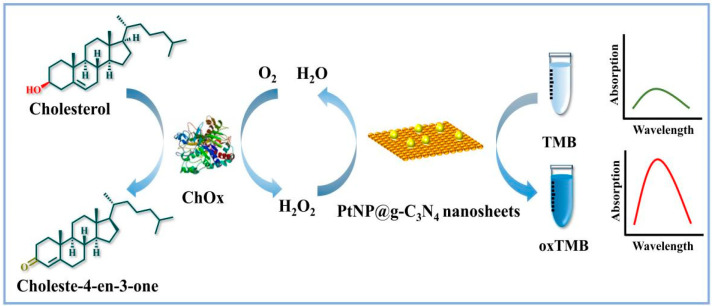
Illustration of constructing PtNP/g–C_3_N_4_ as peroxidase mimic for cholesterol and H_2_O_2_ detection.

In this work, a peroxidase-like hybrid nanocatalyst has been prepared that consists of PtNPs in situ growth on g–C_3_N_4_ nanosheets for H_2_O_2_ and oxidase-based colorimetric assay. Upon combining the excellent catalytic activity of PtNPs with the stability of g–C_3_N_4_ nanosheets, the catalytic performance of the hybrid catalyst such as stability and activity improved remarkably. PtNP@g–C_3_N_4_ nanosheets could quickly catalyze H_2_O_2_ to oxidize TMB, and the absorbance of blue color oxidized TMB (oxTMB) showed a robust linear relationship with the concentration of H_2_O_2_. As proof of the concept for oxidase-based sensing, a colorimetric assay of cholesterol that combined PtNP@g–C_3_N_4_ nanosheets with cholesterol oxidase (ChOx) cascade catalytic reaction was constructed. Cholesterol participates in the formation of cell membrane and can be used as a precursor for vitamin D, steroid hormone, etc. [24,25]. Cholesterol levels are associated with various diseases like cerebral thrombosis, lipid metabolic disorders, etc. [26]. As illustrated in Figure 1, H_2_O_2_ could be generated through the oxidation of cholesterol by cholesterol oxidase, following the PtNP@g–C_3_N_4_ nanosheets could quickly catalyze the oxidation reaction of TMB by the obtained H_2_O_2_, with a proportional production of blue color oxidized TMB (oxTMB). The absorbance of oxTMB showed a robust linear relationship with the concentration of cholesterol. Therefore, a highly sensitive platform for H_2_O_2_ and oxidase-based sensing has been developed which possesses great potential utility in the field of biosensing and medical disease detection.

## 2. Results and Discussion

### 2.1. Characterization of PtNP@g–C_3_N_4_ Nanosheets

**Figure 2:**
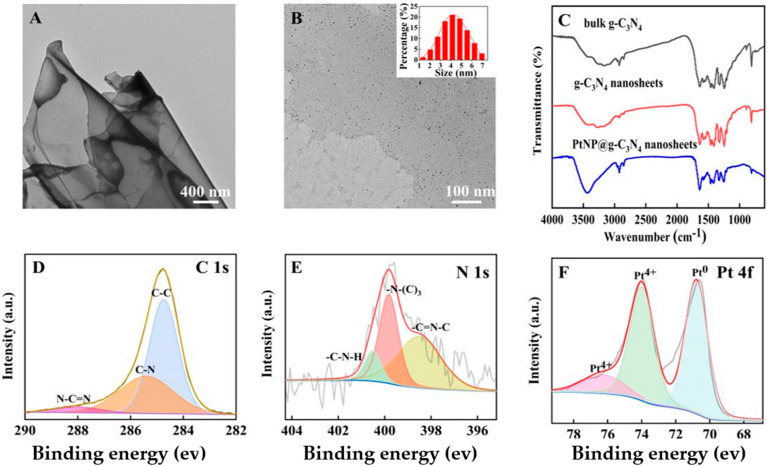
(**A**) The TEM image of prepared g–C_3_N_4_ nanosheets; (**B**) The TEM image of PtNP@g–C_3_N_4_ nanosheets; (**C**) FT–IR spectra of three g–C_3_N_4_ products; (**D**) C 1s spectra of PtNP@g–C_3_N_4_ nanosheets through high–resolution XPS; (**E**) N 1s spectra of PtNP@g–C_3_N_4_ nanosheets through high-resolution XPS; (**F**) Pt 4f spectra of PtNP@g–C_3_N_4_ nanosheets through high-resolution XPS.

It could be observed in the TEM image (Figure 2A) that the g–C_3_N_4_ nanosheets were composed of thin layers with nanometer thickness. Meanwhile, the black dots (which represented platinum particles, about 4.3 nm diameter) were evenly loaded on g–C_3_N_4_ nanosheets (Figure 2B). These obtained images indicated the successful synthesis of PtNP@g–C_3_N_4_ nanosheets.

Further, the FT-IR spectra were scanned. The similarity (Figure 2C) of three g–C_3_N_4_ products indicated that nitric acid etching and PtNPs doping did not change the chemical structure of g–C_3_N_4_ materials. The 810 cm^−1^ sharp peak was caused by the out-of-plane bending vibration of the tri-s-triazine ring, and the series of peaks at 1200–1700 cm^−1^ were due to the stretching vibration of the aromatic CN heterocyclic unit. The peak at 3000~3600 cm^−1^ was caused by the vibration of the amine group or OH group [27].

At last, the chemical composition and elemental state of PtNP@g–C_3_N_4_ nanosheets were studied through high-resolution XPS. The C 1s spectrum was fitted into three peaks (Figure 2D): the 288.0 eV peak was attributed to N-C=N, 285.4 eV to C-N, and 284.6 eV to C-C, respectively. The N 1s spectrum (Figure 2E) could also be divided into three peaks, a peak at 400.5 eV corresponded to C-N-H, 399.8 eV to N-(C)3 and 398.5 eV to C=N-C, respectively. The divided peak of Pt 4f (Figure 2F) at 70.8 eV was attributed to Pt^0^ formed by NaBH4 reduction of Pt^4+^. Other peaks at 74.0 eV and 76.2 eV indicated the valence between divalent and tetravalent.

### 2.2. Catalytic Performance of PtNP@g–C_3_N_4_ Nanosheets

**Figure 3:**
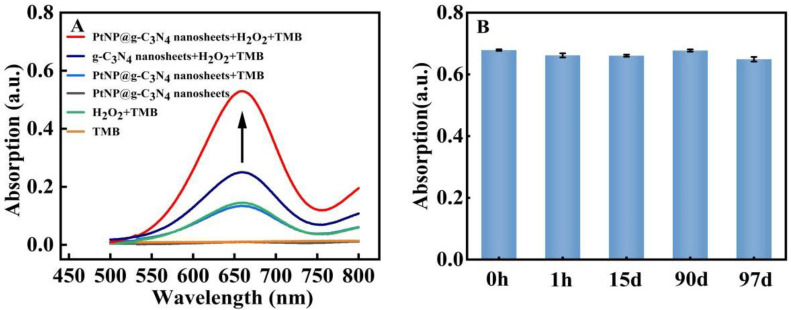
Catalytic activity and stability exploration of PtNP@g–C_3_N_4_ nanosheets (**A**) The absorption spectra of various system; (**B**) The peak absorption of the system with PtNP@g–C_3_N_4_ nanosheets as catalysts in various times.

Peroxidase-mimic activity in our prepared PtNP@g–C_3_N_4_ nanosheets was explored through their catalytic capability to TMB by H_2_O_2_. The absorption peak at 662 nm originated from oxTMB. The existence of PtNP@g–C_3_N_4_ nanosheets could make the 662 nm absorption peak much higher (more than 0.5) than the system without PtNP@g–C_3_N_4_ nanosheets (only about 0.1, Figure 3A). While in the case of only PtNP@g–C_3_N_4_ nanosheets (without TMB and H_2_O_2_), the absorption of the system was nearly zero. In addition, the absorption of the system with PtNP@g–C_3_N_4_ nanosheets as catalyst (more than 0.5) was higher than in the case of g–C_3_N_4_ nanosheets (about 0.25, Figure 3A), which proved that the loading of PtNPs efficiently increased the peroxidase-mimic catalytic activity of g–C_3_N_4_ nanosheets. More importantly, the catalytic performance of PtNP@g–C_3_N_4_ nanosheets was stable. As shown in Figure 3B, it can still maintain good catalytic performance for more than 90 days.

### 2.3. Kinetic Parameters of PtNP@g–C_3_N_4_ Nanosheets as Peroxidase Mimics

**Figure 4:**
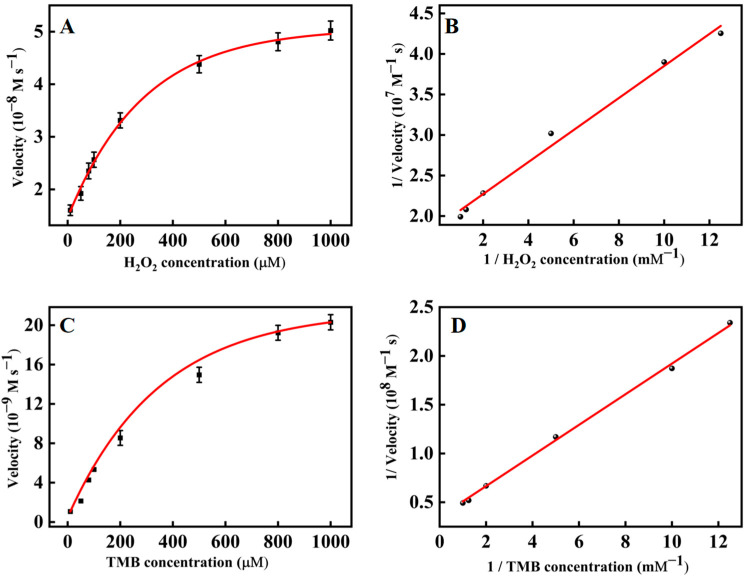
The steady–state kinetics assay (**A**) Michaelis-Menten curve of H_2_O_2_ (TMB concentration was 1 mM); (**B**) Lineweaver-Burk plots with H_2_O_2_ as substrate; (**C**) Michaelis–Menten curve of TMB (H_2_O_2_ concentration was 0.1 mM); (**D**) Lineweaver-Burk plots with TMB as substrate.

The steady-state kinetics of PtNP@g–C_3_N_4_ nanosheets was explored to investigate their catalytic activity. By changing the concentration of one substrate and keeping the alternative constant, the kinetic data was obtained and in good agreement with the Michaelis-Menten equation (Figure 4A,C). Furthermore, a typical Lineweaver-Burk diagram (Figure 4B,D) was constructed through the double-reciprocal method, which obtained the Michaelis constant (*K_m_*) and maximum initial velocity (*V_max_*) (Table 1). It turned out that the *K_m_* of PtNP@g–C_3_N_4_ nanosheets to TMB (0.446 mM) and H_2_O_2_ (0.105 mM) were lower than that of other typical peroxidase mimic nanozymes (Table 1), manifesting that PtNP@g–C_3_N_4_ nanosheets owed a higher affinity for the substrate.

### 2.4. Catalytic Mechanism of PtNP@g–C_3_N_4_ Nanosheets

**Figure 5:**
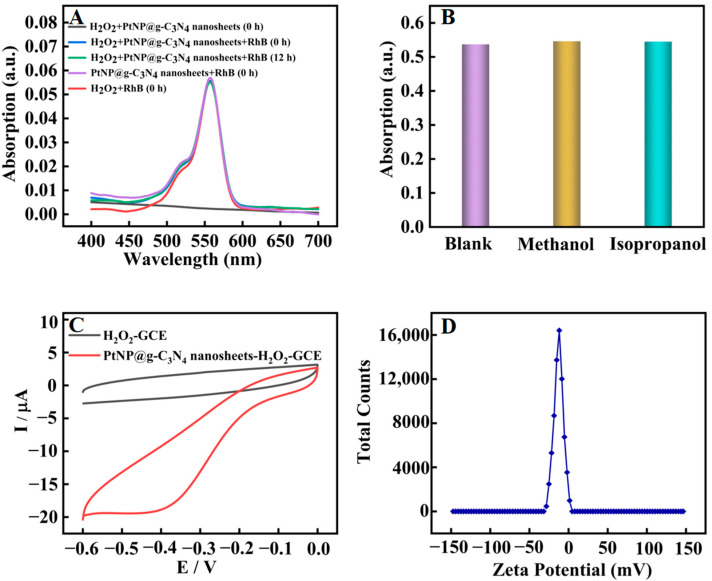
Catalytic mechanism exploration of PtNP@g–C_3_N_4_ nanosheets; (**A**) UV absorption spectra of various system; (**B**) The absorbance value (662 nm) of oxTMB after the addition of methanol or isopropanol; (**C**) Cyclic voltammogram of bare GCE (black line) and PtNP@g–C_3_N_4_ nanosheets modified GCE (Red line) in acetate buffer containing 1 mM H_2_O_2_. Scan rate: 100 mv·s^−1^, platinum electrode as auxiliary, Ag/AgCl electrode as reference electrodes, and the geometric area of glassy carbon electrode used in the electrochemical experiments is 0.07 cm^2^; (**D**) Zeta potential diagram of PtNP@g–C_3_N_4_ nanosheets.

Furthermore, the catalytic mechanism of PtNP@g–C_3_N_4_ nanosheets was explored. Peroxidase mimics usually have two catalytic pathways: the production of hydroxyl radicals (OH•) or the promotion of electron transfer. RhB could react with OH•, resulting in the reduction of its absorption at 550 nm [32]. Firstly, RhB was employed as a high-selective probe to capture and track OH•, which was generated in situ (Figure 5A). The absorbance of RhB almost stayed unchanged for 12 h starting from the addition of H_2_O_2_ and PtNP@g–C_3_N_4_ nanosheets, which proved that OH• was not produced. These results were further supported by UV absorption measurements by using methanol and isopropanol as OH• radical trap (Figure 5B). The addition of methanol or isopropanol had little effect on the absorbance of oxTMB. Later, the electrochemical method was used to investigate the electrocatalytic reduction behavior of our obtained catalyst to H_2_O_2_ (1 mM) in acetate buffer (Figure 5C). The reduction current of PtNP@g–C_3_N_4_ nanosheets modified glassy carbon electrode (GCE) was significantly increased compared with that of bare GCE. These results proved that PtNP@g–C_3_N_4_ nanosheets could increase the electron transfer between GCE and H_2_O_2_ with prominent catalytic ability. Therefore, the peroxidase-mimic activity of our obtained catalyst was probably attributed to the increasing electron transfer effects (the electron that transferred from TMB to H_2_O_2_ was increased by PtNP@g–C_3_N_4_ nanosheets). Moreover, PtNP@g–C_3_N_4_ nanosheets were negatively charged (the potential was −10.2 mV, Figure 5D) so they could adsorb positively charged TMB on their surface through electrostatic interaction, and thus the reaction rate was further promoted.

### 2.5. Optimization of Experiment Conditions for Detection of H_2_O_2_

In order to obtain an ideal analytical performance, the experiment conditions in the detection of H_2_O_2_ and cholesterol were optimized. In this study, *A-A*_0_ was used as a standard to select the optimum condition, *A*_0_ and *A* represented the absorbance responses value of the system with H_2_O_2_ at 0 and 600 μM, respectively, as for cholesterol at 0 and 2 mM.

**Figure 6:**
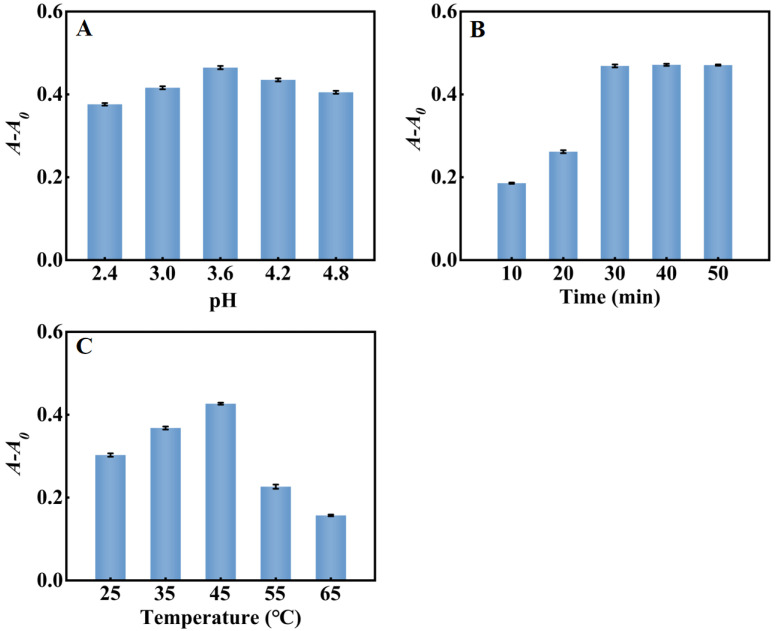
Optimization of the H_2_O_2_ sensing system (**A**) pH; (**B**) reaction time; (**C**) reaction temperature. Error bars indicated the standard deviations of three experiments.

The pH, reaction temperature and reaction time of the system in the detection of H_2_O_2_ were optimized. As shown in Figure 6A, *A-A*_0_ reached the maximum at pH = 3.6. As shown in Figure 6B, *A-A*_0_ of the system increased with time until it reached a balance within 30 min. Consequently, pH = 3.6, 45 °C and a reaction time of 30 min was chosen for subsequent experiments. Figure 6C showed that as the reaction temperature increased from 25 °C to 65 °C, *A-A*_0_ reached the maximum at 45 °C and then decreased gradually, perhaps because PtNP/g–C_3_N_4_ were easily inactivated at a high temperature.

### 2.6. Optimization of Experiment Conditions for Detection of Cholesterol

**Figure 7:**
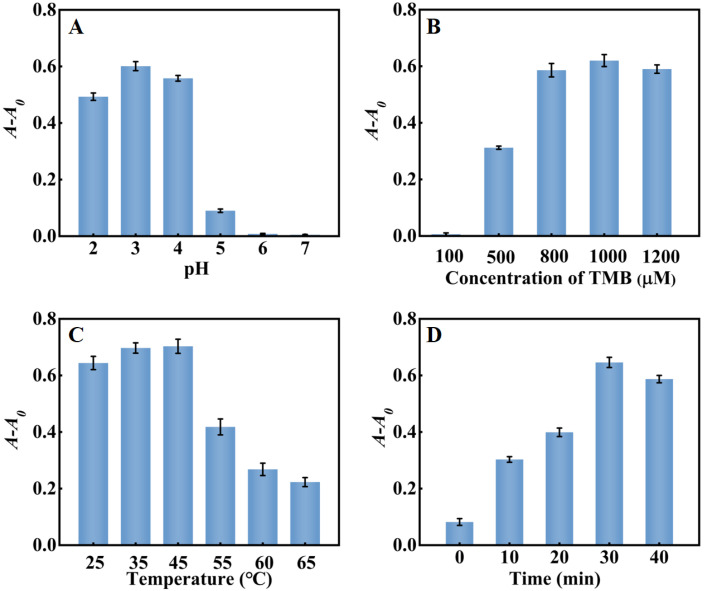
Optimization of system for detection of cholesterol (**A**) pH; (**B**) TMB concentration; (**C**) reaction temperature; and (**D**) reaction time. Error bars indicated the standard deviations of three experiments.

The pH, reaction temperature, TMB concentration, and reaction time of the system in the detection of cholesterol were also optimized. As shown in Figure 7A, *A-A*_0_ reached the maximum at pH = 3. Under strong acid conditions, the decrease in the electron cloud density of nitrogen atoms in TMB would weaken the interaction between PtNP/g–C_3_N_4_ and TMB molecules, inhibiting the catalytic activity of PtNP/g–C_3_N_4_. Conversely, when the pH was higher than 3, unstable H_2_O_2_ tended to decompose into O_2_ and H_2_O, which greatly reduced the amount of H_2_O_2_. The results showed that, similar to natural peroxidase HRP, PtNP/g–C_3_N_4_ exhibited better catalytic activity in the acidic solution. As shown in Figure 7B, with the increase of TMB concentration, *A-A*_0_ gradually increased and then reached the maximum when 1000 μM TMB was used. As shown in Figure 7C, with the increasing of reaction temperature from 25 °C to 65 °C, *A-A*_0_ reached the maximum at 45 °C and then decreased gradually, owing to the fact that cholesterol oxidase and PtNP/g–C_3_N_4_ were easily inactivated at a high temperature. The reaction time also had an effect on the system. As shown in Figure 7D, *A-A*_0_ of the system increased with the reaction time until it reached equilibrium within 30 min. Thus, pH 3.0, 45 °C, 1000 μM TMB, and the reaction time of 30 min were chosen for subsequent cholesterol experiments.

### 2.7. Colorimetric Assay of H_2_O_2_

**Figure 8:**
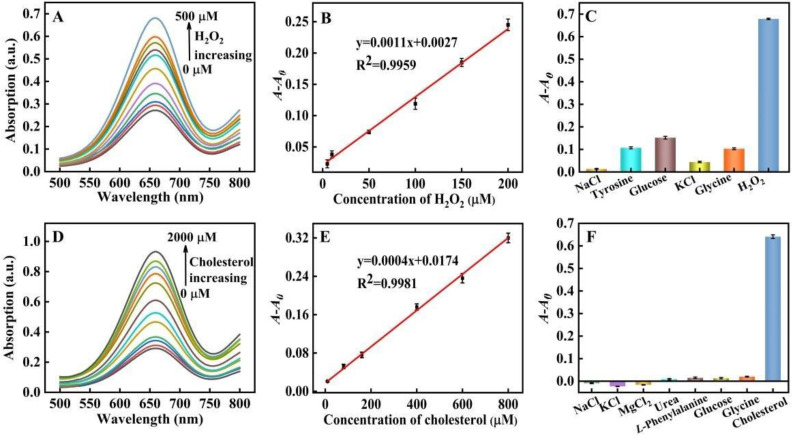
(**A**) UV-visible absorption spectra of the system under various H_2_O_2_ concentrations (0, 5, 10, 50, 100, 150, 200, 250, 300, 400, and 500 μM, respectively); (**B**) The linear relationship between H_2_O_2_ concentration (the concentration from left to right was 5, 10, 50, 100, 150 and 200 μM) and *A-A*_0_; (**C**) The selectivity towards H_2_O_2_ and potential interferents (the concentration was all set at 600 ìM); (**D**) UV-visible absorption spectra under various cholesterol concentrations; (0, 10, 80, 160, 400, 600, 800, 1200, 1400, 1600, 1800, and 2000 μM, respectively); (**E**) The linear relationship between cholesterol concentration and *A-A*_0_. The cholesterol concentration from bottom to top was 10, 80, 160, 400, 600 and 800 μM; (**F**) The selectivity towards cholesterol and potential interferents (the concentrations were all set at 2 mM).

With their peroxidase-mimic catalysis performance, PtNP@g–C_3_N_4_ nanosheets could promote the colorimetric reaction and achieve high assay sensitivity. According to the principle in which H_2_O_2_ assay was first investigated. Figure 8A showed that under the best conditions (the optimization of experiment conditions was in Figure 6), the absorbance value of the system was increased with H_2_O_2_ concentration from 0 to 500 μM. Figure 8B also illustrated a fine linear relationship (R^2^ = 0.9959) between *A-A*_0_ and H_2_O_2_ concentration within the scope 5~200 μM with a low LOD of 3.33 μM (the LOD was estimated according to the equation: LOD = 3σ/S, S: the linear slope in Figure 8B, y = 0.0011x + 0.0027; σ: the standard deviation of blank sample with ten experiments), which was lower than many reports of H_2_O_2_ assays that based on peroxidase-mimic nanozymes (Table 2).

Control experiments were also performed with coexistent NaCl, tyrosine, KCl, glucose, and glycine in blood as potential interfering substances for the evaluation of the selectivity in H_2_O_2_ assay (Figure 8C), H_2_O_2_ or interfering substances were added to the system with a final concentration of 600 μM. *A-A*_0_ of the system was more than 0.6 in the case of H_2_O_2_, as for interfering substances, which was below 0.15. These results proved the high selectively and application prospects of our proposed method of serum sample detection.

### 2.8. Colorimetric Assay of Cholesterol

On the basis of the fact that the catalytic activity of PtNP@g–C_3_N_4_ nanosheets showed a strong dependence on the concentration of H_2_O_2_, which could be produced during the oxidization of many biological substrates by their specific oxidase, our proposed strategy could be exploited as a platform for oxidases-based colorimetric assay. As a proof of concept, a method for cholesterol detection has been established based on PtNP@g–C_3_N_4_ nanosheets and cholesterol oxidase (ChOx) cascade catalytic reaction.

As shown in Figure 8D,E, the absorbance of our proposed assay system increased with the concentration of cholesterol. The linear detection range for cholesterol was 10~800 μM (R^2^ = 0.9981) with a LOD of 9.35 μM (the LOD was estimated according to the equation: LOD = 3σ/S, S: the linear slope in Figure 8E, y = 0.0004x + 0.0174; σ: the standard deviation of blank sample with ten experiments), which reached or was lower than many reported cholesterol assays that also depended on peroxidase mimic nanozymes (Table 3).

Control experiments were carried out with coexistent inorganic salts, urea, L-phenylalanine, glucose, and glycine in blood as potential interfering substances. As shown in Figure 8F, when the interfering substances or cholesterol were added to the system with a final concentration of 2 mM, *A-A*_0_ was more than 0.6 in case of cholesterol, nevertheless *A-A*_0_ of the system containing interfering substances was nearly zero. These results proved the high selectivity of our proposed method, which showed the application prospects for serum sample detection.

### 2.9. Assay of Cholesterol in Fetal Bovine Serum

**Figure 9:**
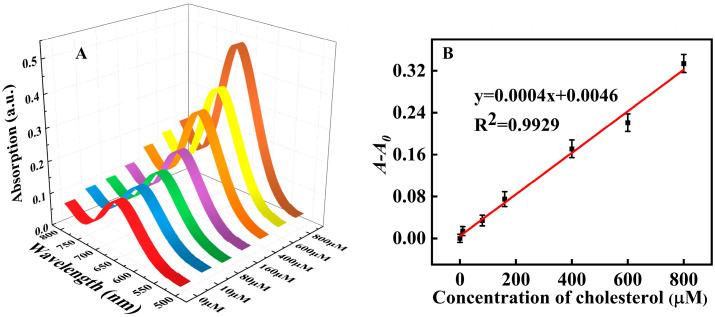
Detection of cholesterol in fetal calf serum; (**A**) UV-visible absorption spectra with various cholesterol concentrations added to solution that contained 10% fetal calf serum; (**B**) The linear relationship between cholesterol concentration and *A-A*_0_.

Our proposed platform was further used in the cholesterol analysis of fetal bovine serum to testify for its application for biological samples. Different standard concentrations of cholesterol were added into the solution that contained 10% fetal calf serum. As shown in Figure 9, the absorbance value increased in proportion to the cholesterol concentration, and a good linear relationship has been found between the cholesterol concentration and *A-A*_0_ (R^2^ = 0.9917). These results showed that our established platform has a certain application prospect in clinical medical detection.

## 3. Materials and Methods

### 3.1. Materials and Instruments

Cholesterol (≥99%) was purchased from Shanghai Yuanye Biotechnology Co., Ltd. (Shanghai, China). Fetal bovine serum (FBS) was purchased from Zhejiang Tianhang Biotechnology Co., Ltd. (Huzhou, China). 3,3′,5,5′-tetramethylbenzidine (TMB, ≥99.7%), glycine (≥99), l-phenylalanine (≥99%), melamine (99%), urea (≥99.5%), dimethyl sulfoxide (DMSO, 95%), glucose (AR), and polyvinylpyrrolidone (PVP, 95%) were purchased from Shanghai Aladdin Biochemical Technology Co., Ltd. (Shanghai, China). Cholesterol oxidase (ChOx, ≥95%), Rhodamine B (RhB, AR), and chloroplatinic acid hexahydrate (H_2_PtCl_6_.6H_2_O, AR) were obtained from Shanghai Macklin Biochemical Co., Ltd. (Shanghai, China). Polyethylene glycol mono-4-octylphenyl ether (Triton X-100) was provided by Saen Chemical Technology (Shanghai, China) Co., Ltd. All the aqueous solutions in this study were prepared with ultrapure water (>18.2 MΩ·cm, Millipore-D 24uv).

UV-vis absorption spectra were collected by TU-1901 UV-visible spectrophotometer (Beijing, China). The morphologies were performed on a Hitachi HT-7700 transmission electron microscope (TEM, Tokyo, Japan). Fourier transform infrared spectra (FT-IR) were recorded on a Thermo Scientific Nicolet iS10 (Waltham, MA, USA). X-ray photoelectron spectroscopy (XPS) measurement was measured by Thermo Scientific Escalab 250Xi (Waltham, MA, USA). Zeta-potential analysis was obtained from Anton Paar. Electrochemical data was conducted at CHI 660E electrochemical workstation (Shanghai, China).

### 3.2. Synthesis of PtNP@g–C_3_N_4_ Nanosheets

First, the bulk g–C_3_N_4_ was prepared according to our previous method [41]. After 2 h ultrasonic treatment in 100 mL 5 M nitric acid, 1 g bulk g–C_3_N_4_ was refluxed for 24 h under 120 °C. The product was then washed to neutrality with ultrapure water through 5 min centrifugation at 2680× *g* repeatedly. After thorough drying, 200 mg power was again dispersed in 40 mL ultrapure water and was treated with ultrasound for 2 h. Then the dispersive g–C_3_N_4_ nanosheets were obtained.

PtNP@g–C_3_N_4_ nanosheets (3 mg/mL g–C_3_N_4_ nanosheets included) were synthesized as follow: After 0.22 µm filter dialysis to remove the large size nanosheets, the 1 mL g–C_3_N_4_ nanosheets solution was mingled with 7 mL polyvinylpyrrolidone (PVP, 16 mg) solution, and 70 µL 0.1 M H_2_PtCl_6_.6H_2_O, followed with heating at 95 °C for 20 min. After that 500 µL freshly prepared 150 mM NaBH_4_ was dropped into the above solution continued with stirring at 95 °C for 30 min. Lastly, after centrifugation and washing with ultrapure water to neutrality, the obtained precipitate was ultrasonically treated in 1 mL ultrapure water and then kept at 4 °C in refrigerator, with ultrasonic treatment for 10 min before use.

### 3.3. Investigation of PtNP@g–C_3_N_4_ Nanosheets Peroxidase-Mimic Activity

We investigated the catalytic activity of PtNP@g–C_3_N_4_ nanosheets as below: 5 μL obtained (3 mg/mL g–C_3_N_4_ nanosheets included) PtNP@g–C_3_N_4_ nanosheets, 50 μL of 1 mM H_2_O_2_, 50 μL of 1 mM TMB, and 150 μL of 0.2 M pH 3.0 acetate buffer were mixed in a tube with a final volume of 500 μL. After incubation at 35 °C for 40 min, the absorbance of the mixture from 500 to 800 nm was detected through UV-visible spectroscopy.

### 3.4. Steady-State Kinetic Assays

Steady-state kinetic analysis of PtNP/g–C_3_N_4_ peroxidase-like activity was carried out by time scanning spectrum. TMB with varied concentration was added into the tube with 50 μL 1 mM H_2_O_2_, 150 μL acetate buffer (0.2 M, pH 3.0), and 5 μL PtNP/g–C_3_N_4_ mixed, and the absorbance value of the mixture following the change of time (2 min) was measured at a maximum absorption wavelength. The reaction speed of the system with different TMB concentrations could be obtained by the Lambert-Beer law (*A* = *εbc*, A represents the measured absorbance value, the molar absorption coefficient *ε* = 39,000 mol^−1^cm^−1^, the thickness of absorption layer *b* = 0.2 cm, *c* is the concentration of the light-absorbing substance) and the Michaelis-Menten equation 1*/V* = (*K_m_/V_max_*) × 1/[*S*] + 1/*V_max_* (*V*, [*S*], *V_max_* and *K_m_* represents initial rate, substrate concentration, the maximum reaction rate, and the Michaelis constant, respectively) could be curved, with *K_m_* and *V_max_* being calculated through the equation. Subsequently, according to the same process, different concentrations of H_2_O_2_ were added to a mixture containing 5 μL PtNP/g–C_3_N_4_, 50 μL 10 mM TMB, and 150 μL acetate buffer, and *K_m_* and *V_max_* could also be calculated.

### 3.5. H_2_O_2_ Colorimetric Assay

In the colorimetric detection of H_2_O_2_, 100 μL H_2_O_2_ with various concentration, 10 μL PtNP@g–C_3_N_4_, 50 μL 10 mM TMB, 150 μL acetic acid buffer (0.2 mol/L, pH = 3.0), and 190 μL ultrapure water were mixed in a tube. After that the tube was incubated at 45 °C for 10 min and then the absorbance value of the mixture was detected.

### 3.6. Cholesterol Colorimetric Assay

As for the detection of cholesterol, 20 μL cholesterol with different concentration, 10 μL 100 U/mL ChOx and 265 μL ultrapure water were mixed in a tube, which was then incubated at 37 °C for 30 min. Later, 5 μL PtNP@g–C_3_N_4_, 50 μL 10 mM TMB, 150 μL acetic acid buffer (0.2 mol/L, pH = 3.0) was mixed with the solution, continued by a 30 min incubation at 45 °C. Finally, UV-visible spectroscopy was used to measure the absorbance value of the mixture.

## 4. Conclusions

In summary, we have fabricated a hybrid catalyst through PtNPs in situ growth on g–C_3_N_4_ nanosheets with increased peroxidase-mimic preformance. Based on their peroxidase-like capability to catalyze the oxidation of TMB, PtNP@g–C_3_N_4_ nanosheets have been used to develop a sensitive colorimetric strategy for H_2_O_2_ assay (LOD: 3.33 μM). More meaningfully, a colorimetric platform based on this strategy can be exploited for assay of many biological substrates where H_2_O_2_ can be generated or consumed based on specific oxidase-catalyzed oxidation. As a proof of concept, a quantitative colorimetric assay for cholesterol (LOD: 9.35 μM) was conducted based on PtNP@g–C_3_N_4_ nanosheets and cholesterol oxidase cascade catalytic reaction, which is more sensitive than many reported methods that depended on peroxidase-mimic nanozymes. The PtNP@g–C_3_N_4_ nanosheets based detection platform also exhibits high selectivity. Moreover, it works well in serum samples, indicating the promising potential applications in the clinical diagnosis of biomolecules related to H_2_O_2_. We plan to develop composite materials based on g–C_3_N_4_ nanosheets with excellent catalytic performance and apply it to biochemical analysis.

## Figures and Tables

**Table 1:** Comparison of the *K_m_* and *V_max_* of Pt/GCNS nanocomplex, HRP, and other typical peroxidase mimic nanozymes.

	*K_m_* (mmol/L)		*V_max_* (1 × 10^−8^ M s^−1^)		
Catalysts	TMB	H_2_O_2_	TMB	H_2_O_2_	Reference
^a^ NGZF	0.907	115.52	9.71	7.44	[28]
^b^ MoS_2_	0.825	2.083	1.161	1.346	[6]
^c^ N-doped TiO_2_	0.45	0.72	11.5	6.8	[29]
^d^ CuNCs	0.648	29.16	5.96	4.22	[30]
Hemin	4.26	2.95	1.108	0.637	[31]
PtNP/g–C_3_N_4_	0.446	0.105	2.84	5.33	This work

^a^ NGZF: N-doped graphene/ZnFe_2_O_4_; ^b^ MoS_2_: MoS_2_ nanosheets; ^c^ N-doped TiO_2_: nitrogen doped titania nanoparticles; ^d^ CuNCs: copper nanoclusters.

**Table 2:** The detection limits and linear ranges of H_2_O_2_ detection methods in different catalyst systems.

Method	Material	Linear Range (mmol/L)	LOD (μmol/L)	Reference
Colorimetry	CoAl-^a^ ELDHs	0.01~0.2	10	[33]
Amperometry	^b^ CoOOH NSs	0~1.6	40	[34]
Colorimetry	DNA/CuAl-^c^ LDHs	0.02~2.0	10	[35]
Amperometry	^d^ AgNPs /TWEEN/GO	0.02~23.1	8.7	[36]
Amperometry	^e^ SCMf-100	0.1~25	15	[37]
Colorimetry	PtNP/g–C_3_N_4_	0.05~0.2	3.33	This work

^a^ ELDHs: exfoliated layered double hydroxides; ^b^ CoOOH NSs: cobalt oxyhydroxide nanosheets; ^c^ LDHs: layered double hydroxides; ^d^ AgNPs/TWEEN/GO: silver nanoparticle-decorated graphene oxide; ^e^ SCMf-100/GCE: spinel cobalt manganese oxides.

**Table 3:** The detection limits and linear ranges of cholesterol detection methods in different catalyst systems.

Method	Material	Linear Range (mmol/L)	LOD (μmol/L)	Reference
Colorimetry	^f^ Por-NiCo_2_S_4_	0.1~9.0	19.36	[24]
Amperometry	Chox/Fe_2_O_3_	0.1~8.0	18	[38]
Colorimetry	^g^ Au/MoS_2_	0.04~1.0	15	[39]
Amperometry	^h^ AgNPs/GCE	0.1~20.0	25.8	[40]
Colorimetry	PtNP/g–C_3_N_4_	0.01~0.8	9.35	This work

^f^ Por-NiCo_2_S_4_: porphyrin functionalized NiCo_2_S_4_ yolk-shell nanospheres; ^g^ Au/MoS_2_: gold nanoparticles supported on MoS_2_ nanoribbons; ^h^ AgNPs/GCE: silver nanoparticles modified glassy carbon electrode.

## Data Availability

Not applicable.

## Sample Availability

Samples of the compounds are not available from the authors.

## Abbreviations

PtNPs: Pt nanoparticles; PtNP/g–C_3_N_4_ nanosheets, Pt nanoparticles loaded graphitic carbon nitride nanosheets; TMB, 3,3′, 5,5′-tetramethylbenzidine; HRP, horseradish peroxidase; DMSO, dimethyl sulfoxide; PVP, polyvinylpyrrolidone; ChOx, cholesterol oxidase; RhB, Rhodamine B; H_2_PtCl_6_·6H_2_O, chloroplatinic acid hexahydrate; Triton X-100, polyethylene glycol mono-4-octylphenyl ether; TEM, transmission electron microscope; XPS, X-ray photoelectron spectroscopy; FT-IR, Fourier transform infrared spectra.
